# Case of Malposition of the Bladder

**Published:** 1871-06

**Authors:** E. Dooley

**Affiliations:** Selma, Ill.


Article VII.—*Case of Malposition of the Bladder.* By E. Dooley, M.D., Selma, Ill.

March 23d, 1871, was called hurriedly in the night, four miles out, to attend Mrs. McM., in her fifth confinement. Previous labors had been very short—"never could get a doctor in time." Found patient in hard labor, worrying about its tediousness.

On examination, found os fully dilated, with brow presentation. With strong uterine contractions, delivery was soon effected, and the rousing cry of a large, fine looking boy greeted our ears. But on examination, I discovered, on the lower, or coccygeal portion of the spine, a ruptured, encysted tumor, with a heavy, corrugated, scrotum-like covering, excepting a space of about an inch in length and one-fourth as wide, elliptical in shape, about the middle of the tumor. This space was covered with mucous membrane. At this point the tumor was ruptured, probably during the transit through the maternal organs, and the contents had escaped. The legs were firmly flexed, heels almost touching the buttocks, and very rigid and immovable. Diagnosis, spinal tumor.

Saw the little sufferer dressed, amidst his piercing and continuous cries, prognosticated unfavorably, and left.

Called the next evening, and found the poor little unfortunate still crying, and was told it had never ceased, although paregoric had been given freely. On inquiring for the usual evacuations, was told that its "bowels had moved," but it had "made no water." Was disposed to question this report, as I was quite sure I could detect the oder of urine. Penis, scrotum, and anus apparently normal, but, examining closely, found the penis impervious. Turning again to the formerly collapsed but now partially filled tumor, found a dark, amber-colored fluid dribbling away from its ruptured orifice, with the unmistakable odor of urine. Concluded this must be the bladder thus curiously located on this otherwise well developed infant.

It lived three days, and then sank, exhausted by convulsions and unceasing pain, consequent upon the excoriations and inflammation within and around the tumor or bladder.

